# Natural Products in Modulating Methamphetamine-Induced Neuronal Apoptosis

**DOI:** 10.3389/fphar.2021.805991

**Published:** 2022-01-04

**Authors:** Yiwei Zeng, Yunhui Chen, Su Zhang, Huan Ren, Jialin Xia, Mengnan Liu, Baozhi Shan, Yulan Ren

**Affiliations:** ^1^ College of Acupuncture-moxibustion and Tuina, College of Basic Medicine, College of Nursing, College of Chinese Classics, Chengdu University of Traditional Chinese Medicine, Chengdu, China; ^2^ Traditional Chinese Medicine Hospital Affiliated to Southwest Medical University, Luzhou, China; ^3^ School of Humanities, Jiangxi University of Traditional Chinese Medicine, Nanchang, China

**Keywords:** methamphetamine, dopaminergic neurons, apoptosis, natural products, neurodegenaration

## Abstract

Methamphetamine (METH), an amphetamine-type psychostimulant, is highly abused worldwide. Chronic abuse of METH causes neurodegenerative changes in central dopaminergic neurons with numerous neuropsychiatric consequences. Neuronal apoptosis plays a critical role in METH-induced neurotoxicity and may provide promising pharmacological targets for preventing and treating METH addiction. In recent years, accumulating evidence has revealed that natural products may possess significant potentials to inhibit METH-evoked neuronal apoptosis. In this review, we summarized and analyzed the improvement effect of natural products on METH-induced neuronal apoptosis and their potential molecular mechanisms on modulating dopamine release, oxidative stress, mitochondrial-dependent apoptotic pathway, endoplasmic reticulum stress-mediated apoptotic pathway, and neuroinflammation. Hopefully, this review may highlight the potential value of natural products in modulating METH-caused neuronal apoptosis and provide useful information for future research and developments of novel and efficacious pharmacotherapies in this field.

## Introduction

Methamphetamine (METH), also known as “ice” or “crystal,” is one of the most widely used and abused illicit amphetamine-type stimulants (Courtney and Ray, 2014; Jones et al., 2020). Statistics reveal that in 2019, approximately 27 million people (0.5% of the global population) reported using METH (UNODC, 2020). The number of METH abusers in China and the United States reached an estimated 1.18 million (out of 2.14 million drug users) and 1.7 million (more than half of them suffering METH use disorder), respectively (CNNCC, 2019; Elinore, 2020). Studies also demonstrate that METH administration is highly prevalent in Australia, Canada, and South Africa (Australian Institute of Health and Welfare, 2020; Bach et al., 2020; Sorsdahl et al., 2021). This highly addictive stimulant drug causes serious societal and public health concerns globally, and considerable attention and effort need to be devoted to researching and developing effective intervention strategies against METH-evoked neurotoxic consequences.

Epidemiological and clinical studies have demonstrated that METH is a potent central nervous system stimulant and can significantly impact the human body physically, behaviorally, cognitively, and psychiatrically (Schep et al., 2010; Kevil et al., 2019). Acute METH intake may cause euphoria, delusions, agitations, hypersexuality, or even hyperthermia, cerebrovascular hemorrhages, arrhythmias, heart attacks, and renal failure in high doses (Courtney and Ray, 2014; Glasner-Edwards and Mooney, 2014; Baradhi et al., 2019; Kevil et al., 2019). Long-term METH abuse elicits neuropsychiatric impairments, including cognitive decline, altered decision-making, psychosis, and psychomotor dysfunction (Krasnova and Cadet, 2009; Dean et al., 2013; Lappin et al., 2018; Mizoguchi and Yamada, 2019). Its withdrawal symptoms in abstinence include depression, agitation, fatigue, and intense craving (Krasnova and Cadet, 2009; Feng et al., 2020). These neuropsychiatric complications are mostly attributed to METH-induced neurotoxicity. Animal-based studies and human neuroimaging analysis have revealed that repeated use of METH can induce neurodegeneration of dopaminergic and serotoninergic terminals in the striatum, cortex, and hippocampus and primarily affects the nigrostriatal dopaminergic pathway (Ernst et al., 2000; Friend and Keefe, 2013; McFadden et al., 2018; Golsorkhdan et al., 2020). METH-evoked neurotoxicity may include direct damage to terminals, oxidative stress, mitochondria dysfunction, neuronal excitotoxicity, endoplasmic reticulum stress, and neuroinflammation, leading to dopaminergic neuron apoptosis (Jayanthi et al., 2004; Yang et al., 2018; Kim et al., 2020). Although substantial progress has been made in deciphering the mechanisms of METH-induced neuronal apoptosis, currently effective pharmacotherapies for METH addiction remain scarce (Yang et al., 2018). Hence, exploration of natural products as potential interventions against METH-elicited complications has become a research hotspot in recent years (Moszczynska and Callan, 2017; Chiang et al., 2019).

Natural products are bioactive components from natural organisms, including plants, animals, insects, marine organisms, microorganisms (Xie et al., 2021). These agents have been widely used by human societies for thousands of years (Baker et al., 2007), and their bioactivities are of great interest to enormous research fields (Ekiert and Szopa, 2020). In recent decades, a growing body of research has demonstrated that natural products exert anti-apoptotic effects on METH-induced neurotoxicity and may yield promising targets for further investigation of effective pharmacological interventions. Converging lines of evidence suggest that natural products can attenuate symptoms of addiction and drug use disorders and modulate the complex mechanisms underlying METH-evoked apoptosis in various ways (Lu et al., 2009). Moreover, METH users have been reported to have an increased risk of developing Parkinson’s disease. Natural products may garner considerable neuroprotective properties and effectively mitigate neurodegeneration of Parkinson’s disease that partially shares pathogenesis with METH-induced apoptosis, including dopamine release, oxidative stress, and mitochondria damage (Rios et al., 2016; Solayman et al., 2017). Their application may merit further research and development as a promising strategy for pharmacological intervention against METH-induced neuronal apoptosis. Hence, to provide useful insights for future research and development of novel and efficacious pharmacotherapies in this field, a systematic and comprehensive review is of necessity.

In this review, electronic databases, including PubMed, EMBASE, Web of Science, Cochrane Library, China National Knowledge Infrastructure, China Biomedical Literature Database, Wanfang Database, and Chinese Scientific Journals Database, were searched from the inception to August 2021 to identify eligible studies. All the included individual studies were assessed according to the Best practice in research—Overcoming common challenges in phytopharmacological research (Heinrich et al., 2020). The anti-apoptotic effects of natural products on METH-evoked neurotoxic consequences through mediating multiple pathways were summarized and analyzed, including dopamine release, oxidative stress, mitochondrial-dependent apoptotic pathway, endoplasmic reticulum stress-mediated apoptotic pathway, and neuroinflammation. The detailed information of natural products and their potential effects with mechanisms on modulating METH-evoked neuronal apoptosis are illustrated in Tables 1, 2.

**TABLE 1:** Anti-apoptotic activities of natural products in METH-induced neuronal apoptosis.

Extracts/Monomers	Cells/Animal	Dosage	Related mechanisms	Detailed pathways	Refs
Ginseng total saponin	Male Swiss-Webster mice	50, 100 mg/kg	DA release	Blocking DA uptake inhibiting degradation of DA by MAO	Oh et al. (1997a); Oh et al. (1997b)
Limonene	Male Sprague-Dawley rats and ICR mice	200, 400, 600 mg/kg	DA release	Increasing GABA levels through activating GABA B receptors	Yun (2014)
Apocynin	Male Sprague–Dawley rats	0.01–100 μM	DA release	Not conclusive	Miller et al. (2014)
	Male ICR mice	50 mg/kg daily for 7 days	DA release	Inhibiting ERK-dependent p47phox activation	Dang et al. (2016)
			Oxidative stress		
			Neuroinflammation		
Pseudoginsenoside-F11	Male ICR mice	8 mg/kg daily for 5 days	DA release	Regulating GABAergic neurons and μ-opioid receptors.	Li et al. (2000)
	Male Wistar rats	3, 6 mg/kg for 12 days	Oxidative stress	Increasing SOD and GSH	Chen et al. (2016)
				Decreasing MDA	
Ginsenoside RE	C57BL/6 mice	10, 20 mg/kg, p.o., twice a day	Oxidative stress	Inactivating PKCδ	Shin et al. (2014)
			Neuroinflammation	Inducing GPx activity	
Resveratrol	Dopaminergic neurons of mice	20 μM	Oxidative stress	Decreasing ROS production	Sun et al. (2015)
			ERS	Decreasing intracellular Ca^2+^ concentration	
	C57BL/6 mice	10, 100 mg/kg	Oxidative stress	Activating the Keap1-Nrf2 pathway	Zeng et al. (2021)
	N27 dopaminergic cells	10 μM	Mitochondria-dependent pathway	Blocking caspase-3 activation	Kanthasamy et al. (2011)
Crocin	Male Wistar rats	30, 60, 90 mg/kg	Oxidative stress	Increasing SOD and GSH	Shafahi et al. (2018); Mozaffari et al. (2019)
			Neuroinflammation	Decreasing MDA and TNF-α	
				Regulating CREB-BDNF signaling pathway	
Epigallocatechin gallate	CD-1 male mice	2 mg/kg	Oxidative stress	Increasing GPx-4 protein	Pan et al. (2020)
				Decreasing SOD-1 protein	
TCPE	PC12 cells	6.25–400 μg/ml	Oxidative stress	Increasing SOD and GPx	Zeng et al. (2020)
				Decreasing ROS and MDA	
Curcumin	Male Wistar rats	100, 200 mg/kg for 7 days	Oxidative stress	Increasing SOD and GPx	Hadizadeh-Bazaz et al. (2021)
			Neuroinflammation	Decreasing TNF-α and MDA	
Astragaloside IV	Male Wistar rats	10, 20 mg/kg	Mitochondria-dependent pathway	Increasing Bcl-2	Lu et al. (2015)
				Decreasing caspase-3	
Asiatic acid	SH-SY5Y cells	20 μM	Neuroinflammation	Decreasing TNF-α and IL-6	Park et al. (2017)
			Mitochondria-dependent pathway	Inhibiting translocation of NF-κB/STAT3 and ERK phosphorylation	
				Inhibiting cleavage of procasepase-3	
Antrodia camphorata enzymes	PC12 cells	62.5, 125, 250 μg/ml	Mitochondria-dependent pathway	Inhibiting activity of caspase-3	Chi et al. (2017)
			Oxidative stress	Decreasing ROS production	
Gastrodin	Cortical neurons of SD rats	25 mg/L	Mitochondria-dependent pathway	Decreasing caspase-3	Ma et al. (2020); Shin et al. (2011); Yang et al. (2020)
				Regulating CREB-BNDF signaling pathway	
Epicatechin	HT22 hippocampal cells	10–20 μM	ERS	Down-regulating CHOP, ROS, caspase-3, -8, -9, PARP	Kang et al. (2019)
Aromadendrin	SH-SY5Y Cells	10–40 μM	ERS	Decreasing CHOP, Bax; Increasing bcl-2; Inhibiting mTOR phosphorylation	Lee et al. (2021)
			P13K/Akt/mTOR pathway		

DA, dopamine; MAO, monoamine oxidase; GABA, gamma-aminobutyric acid; ERK, extracellular signal-regulated kinase; SOD, superoxide dismutase; GSH, glutathione; MDA, malondialdehyde; PKCδ, protein kinase C-δ; GPx, glutathione peroxidase; ROS, reactive oxygen species; ER, endoplasmic reticulum; CHOP, TNF-α, tumor necrosis factor-α; CREB, cyclic AMP response element binding protein; BNDF, brain-derived neurotrophic factor; Keap-1, Kelch-like ECH associated protein-1; Nrf2, nuclear factor erythroid 2-related factor 2; Bcl-2, B-cell lymphoma 2; IL-6, interleukin 6; TCPE, terminalia chebula polyphenol extract; NF-κB, nuclear factor kappa B; STAT3, signal transducer and activator of transcription proteins 3; PARP, poly ADP-ribose polymerase.

**TABLE 2:** Detailed information and chemical structures of natural products.

Monomers	Systematic name	Origin	Chemical structures
Limonene	(4S)-4-Isopropenyl-1-methylcyclohexene	Fruits *of Citrus L.,* Rutaceae	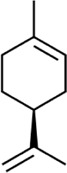
Apocynin	1-(4-Hydroxy-3-methoxyphenyl)-ethanone	*Picrorhiza kurroa* Royle ex Benth*,* Plantaginaceae	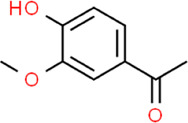
Pseudoginsenoside-F11	(3beta,6alpha,12beta,24R)-3,12,25-Trihydroxy-20,24-epoxydammaran-6-yl 2-O-(6-deoxy-alpha-L-mannopyranosyl)-beta-D-mannopyranoside	*Panax quinquefolius L,* Araliaceae	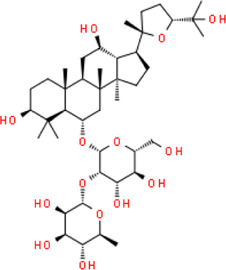
Ginsenoside RE	(3beta,6alpha,12beta)-20-(beta-D-Glucopyranosyloxy)-3,12-dihydroxydammar-24-en-6-yl 2-O-(6-deoxy-alpha-L-mannopyranosyl)-beta-D-glucopyranoside	*Panax ginseng* C. A. Mey., Araliaceae	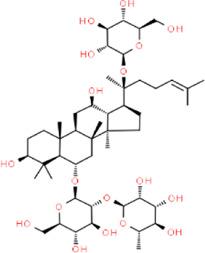
Resveratrol	5-[(E)-2-(4-Hydroxyphenyl)vinyl]-1,3-benzenediol	Red wine	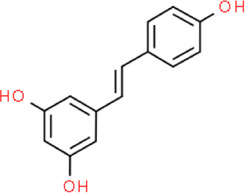
Crocin	Bis [(2S,3R,4S,5S,6R)-3,4,5-trihydroxy-6-({[(2R,3R,4S,5S,6R)-3,4,5-trihydroxy-6-(hydroxymethyl)tetrahydro-2H-pyran-2-yl]oxy}methyl)tetrahydro-2H-pyran-2-yl] (2E,4E,6E,8E,10E,12E,14E)-2,6,11,15-tetramet hyl-2,4,6,8,10,12,14-hexadecaheptaenedioate	*Crocus sativus L.,* Iridaceae	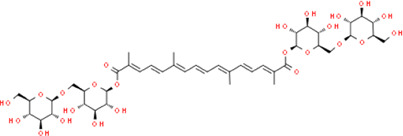
Epigallocatechin gallate	(2R,3R)-5,7-Dihydroxy-2-(3,4,5-trihydroxyphenyl)-3,4-dihydro-2H-chromen-3-yl 3,4,5-trihydroxybenzoate	*Camellia sinensis L.*, Theaceae	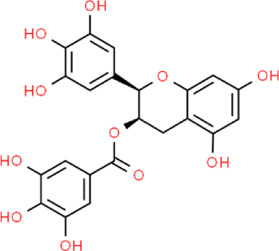
Curcumin	(1E,6E)-1,7-Bis(4-hydroxy-3-methoxyphenyl)-1,6-heptadiene-3,5-dione	*Curcuma longa L.,* Zingiberaceae	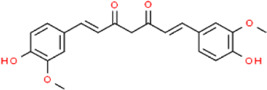
Astragaloside IV	(3beta,6alpha,9beta,16beta,20R,24S)-16,25-Dihydroxy-3-(beta-D-xylopyranosyloxy)-20,24-epoxy-9,19-cyclolanostan-6-yl beta-D-glucopyranoside	*Astragalus mongholicus* bunge, Fabaceae	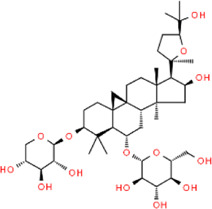
Asiatic acid	(2alpha,3beta)-2,3,23-Trihydroxyurs-12-en-28-oic acid	*Centella asiatica (L.)* Urb, Apiaceae	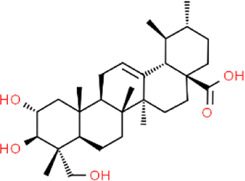
Gastrodin	4-(Hydroxymethyl)phenyl beta-D-glucopyranoside	*Gastrodia elata* Blume, Orchidacea	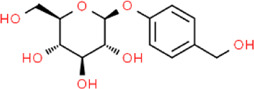
Epicatechin	(2R,3R)-2-(3,4-Dihydroxyphenyl)-3,5,7-chromanetriol	*Camellia sinensis* (*L.*) kuntze, Theaceae	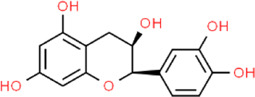
Aromadendrin	(2R,3R)-3,5,7-Trihydroxy-2-(4-hydroxyphenyl)-2,3-dihydro-4H-chromen-4-one	*Abies sibirica* Ledeb. (Syn. *Pinus sibirica* Ledeb)*,* Pinaceae	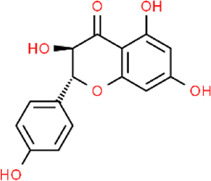

## Main Mechanisms Underlying METH-Induce Neuronal Apoptosis

### METH-Induced Dopamine Release

Dopamine (DA) is a key neurotransmitter associated with the reinforcement effects of drug addiction. METH is a cationic molecule and chiral compound based around a phenylethylamine core (Homer et al., 2008) and can modulate the DA system with a myriad of influences on DA release. It has a high lipid solubility, penetrates the blood-brain barrier readily (Nordahl et al., 2003; Schep et al., 2010), and can be taken up by dopaminergic cells through binding to the dopamine transporter (DAT) due to its chemical similarity with DA (Iversen, 2006). Physiologically, DAT removes DA from the synapse to terminate its neurotransmitter effects (Wang et al., 2015). The binding of METH to DAT causes the blockage of the extracellular DA reuptake and the reverse transportation of DA outside the cell, leading to the increase of DA concentration in the synaptic cleft (Panenka et al., 2013). METH in high concentrations can cross the axons through lipophilic diffusion, exacerbating the accumulation of extracellular DA (Shin et al., 2018). Another primary substrate molecule involved in METH-induced DA release is the vesicular monoamine transporter-2 (VMAT-2), an integral membrane protein transporting DA from the cytosol to synaptic vesicles (Fleckenstein et al., 2009). This transportation is coupled to a vacuolar-type H^+^ pump ATPase. Studies show that METH, as a “weak base,” can disrupt the proton gradient of inner and outer sides of the vesicle required for DA sequestration, eliciting the DA leakage from the vesicle to the cytosol (Schwartz et al., 2006; Panenka et al., 2013). Moreover, METH may induce a decrease of VMAT-2 function and its density on the membrane (Eyerman and Yamamoto, 2005; McFadden et al., 2012). These interactions of METH with DAT and VMAT-2 result in excessive DA accumulation in the intracellular and extracellular surroundings.

### METH-Induced Oxidative Stress and Mitochondria-Dependent Cell Apoptosis

Multiple pathways are involved in the complicated mechanism of METH-caused neurotoxicity, and oxidative stress has been considered an essential contributor to neuronal apoptosis. METH-induced cytosolic and synaptic DA accumulation results in the production of quinone and semi-quinone through autoxidation to generate large amounts of reactive oxygen species (ROS), such as hydrogen peroxide, hydroxyl radicals, and superoxide radicals. Furthermore, a small portion of DA produces H_2_O_2_ with the mediation of monoamine oxidase (MAO) (Hermida-Ameijeiras et al., 2004; McDonnell-Dowling and Kelly, 2017; Yang et al., 2018). METH also inhibits the production of antioxidants, including glutathione, superoxide dismutase (SOD), and catalase (Tata and Yamamoto, 2007; Huang et al., 2013). Thus, the imbalance between ROS and free radical scavengers contributes to the oxidative stress in dopaminergic terminals. Excessive ROS may result in lipids and protein metabolism disruption, mitochondrial dysfunction, and nuclear DNA damage, increasing susceptibility to neuronal damage and death (Potashkin and Meredith, 2006).

Mitochondria is an important site of METH-evoked ROS generation in neural cells. Several investigations have suggested that mitochondria dysfunction plays a crucial role in METH-induced dopaminergic apoptosis. ROS can inhibit key enzymes in the electron transport chain (ETC) of mitochondria, including complex II-III and IV (Wu et al., 2007; Halpin et al., 2014a; Dawson and Dawson, 2017; Moratalla et al., 2017). METH diffuses into mitochondria, and its accumulation disrupts oxidative phosphorylation and suppresses ATP synthesis (Krasnova and Cadet, 2009). In addition, METH inhibits the tricarboxylic acid cycle and ETC, which are essential to ATP production (Barbosa et al., 2015). In turn, the inhibition of ETC enhances ROS production, which may further restrain ETC components through positive feedback, aggravating the dysfunction of mitochondrial metabolism (Yang et al., 2018). Another critical mechanism underlying mitochondria dysfunction triggered by METH is the alteration of mitochondrial dynamics (biogenesis, fusion, fission, and mitophagy) (Michel et al., 2012; Anne Stetler et al., 2013). METH can decrease the expression of certain key molecules related to mitochondrial biogenesis (Valian et al., 2017; Beirami et al., 2018). Mitochondrial dysfunction causes the mitochondrial membrane permeabilization (Kroemer et al., 2007), and subsequently the release of cytochrome-c (Cyt-c), apoptosis-inducing factor (AIF), and Smac/Diablo into the cytosol (Cadet et al., 2003; Jayanthi et al., 2004). METH administration has been shown to up-regulate the expression of pro-apoptotic proteins (Bax, Bak, and Bid) and down-regulate the expression of anti-apoptotic proteins (Bcl-2, Bcl-XL, and BclW) (Jayanthi et al., 2001; Cadet et al., 2003). The increase in the Bax, Bak, and Bid can promote the release of Cyt-c from mitochondria to the cytosol (Roth and D'Sa, 2001). Subsequently, apoptosis protease activating factor 1 (Apaf-1) is activated, initiating the mitochondria-dependent cell apoptosis pathway (the intrinsic pathway) (Cain et al., 2002). Apaf-1 binding to Cyto-c constitutes the “apoptosome,” which serves as a molecular platform for the caspase-9 activation (Jiang and Wang, 2000; Bratton and Salvesen, 2010) and induces sequential caspase-3, -6, and -7 activations (Roth and D'Sa, 2001; Shin et al., 2018). Additionally, several studies have reported that protein kinase C-δ (PKCδ), a member of the PKC family, plays a critical role in the mitochondrial-dependent apoptotic cascade. PKCδ can be proteolytically activated by caspase-3, then translocate to the nucleus mediating DNA fragmentation and apoptosis. In turn, the activated PKCδ enhances caspase-3 production through a positive-feedback loop (Kanthasamy et al., 2003; Kaul et al., 2003).

### METH-Induced Excitotoxicity and Endoplasmic Reticulum Stress

Excitotoxicity refers to the overloaded intracellular calcium levels initiated by excessive glutamate (Glu) release, activating apoptotic pathways, eventually resulting in cellular damage (Halpin et al., 2014b). Glu is the major excitatory neurotransmitter in the brain, and METH has been shown to evoke the excessive release of Glu in the striatum (Stephans and Yamamoto, 1994; Mark et al., 2004). Accumulated Glu binds to amino-5hydroxy-3-methyl-4-isoxazole propionic acid receptors and N-methyl-D-aspartate receptors to initiate downstream signaling pathways, leading to a surge of Ca^2+^ influx (Riddle et al., 2006; Halpin et al., 2014b; Kim et al., 2020). Ca^2+^ is an important intracellular second manager responsible for multiple signal transductions, which can be regulated by the endoplasmic reticulum (ER) through the sequestration of Ca^2+^. METH-induced excess intracellular Ca^2+^ causes the disturbance of intracellular Ca^2+^ concentrations, activates the protein kinases, phosphatase, and nitric oxide synthase (NOS) and eventually results in endoplasmic reticulum stress (ERS) (Bahar et al., 2016; Moratalla et al., 2017). In stress conditions, a decrease in the protein-folding capacity of ER may induce the accumulation of unfolded and misfolded proteins in ER, which causes unfold protein response (UPR) to remove those proteins and maintain ER homeostasis (Go et al., 2017). UPR is mediated by IRE1α, PERK, and ATF6 signaling proteins, triggering UPR-dependent apoptotic signaling by activating C/EBP homologous protein (CHOP) (Sano and Reed, 2013; Bahar et al., 2016). As a transcription factor, CHOP can up-regulate the expressions of Bax and Bak and down-regulate the expressions of Bcl-2 and Bcl-XL to promote apoptosis (Hu et al., 2018). The elevated Ca^2+^ activates the calpain, a cytosolic calcium-activated neutral cysteine endopeptidase. The activated calpain translocates from the cytosol to the membrane, where it cleaves procaspase-12 (Nakagawa and Yuan, 2000), then the active caspase-12 initiates positive feedback to activate caspase-9 and -3 to potentiate apoptosis (Martinez et al., 2010).

### METH-Induced Neuroinflammation

The neuroinflammation induced by METH is closely related to the microglial activation and the production of pro-inflammatory cytokines (Goncalves et al., 2008). Microglial cells monitor neuronal homeostasis, serve as the immune defense against neuronal damage, and can be activated upon neuronal injury and neurotoxic agents (Lull and Block, 2010; Shaerzadeh et al., 2018). METH stimulation may elicit the morphological alteration of microglial cells (Xu et al., 2017). Neuroimaging has demonstrated that chronic METH administration induces microgliosis reaction in humans (Sekine et al., 2008). Activated microglial cells secrete enormous pro-inflammatory cytokines, including interleukin6 (IL-6) and tumor necrosis factor-α (TNF-α), which induce prolonged neuroinflammation (Goncalves et al., 2010). This inflammatory response may be associated with certain signaling pathways, such as nuclear factor kappa B (NF-κB) and signal transducer and transcription (STAT) pathway activator. Research has shown that METH can activate the NF-κB pathway, inducing its transfer from the cytoplasm to the nucleus and promoting the transcription of pro-inflammatory cytokines in microglia (Shah et al., 2012). METH also causes the increase of signal transducer and activator of transcription proteins 3 (STAT3) and its translocation to the nucleus (Zhao et al., 2019), which carries out the IL-6 signaling (Coelho-Santos et al., 2012). Additionally, studies have demonstrated that mitogen-activated protein kinase (MAPK) may play a crucial role in the METH-induced release of inflammatory cytokines (Yan et al., 2014; Park et al., 2017). The chronic neuroinflammation leads to overexpression of PUMA (p53-up-regulated modulator of apoptosis), a pro-apoptotic protein of the Bcl-2 family that drives Cyt-c release from the mitochondria to initiate caspase cascades (Yang et al., 2018).

## Effects and Mechanisms of Natural Products on METH-Induced Apoptosis

### Natural Products Targeting DA Release to Modulate METH-Induced Apoptosis

**FIGURE 1:**
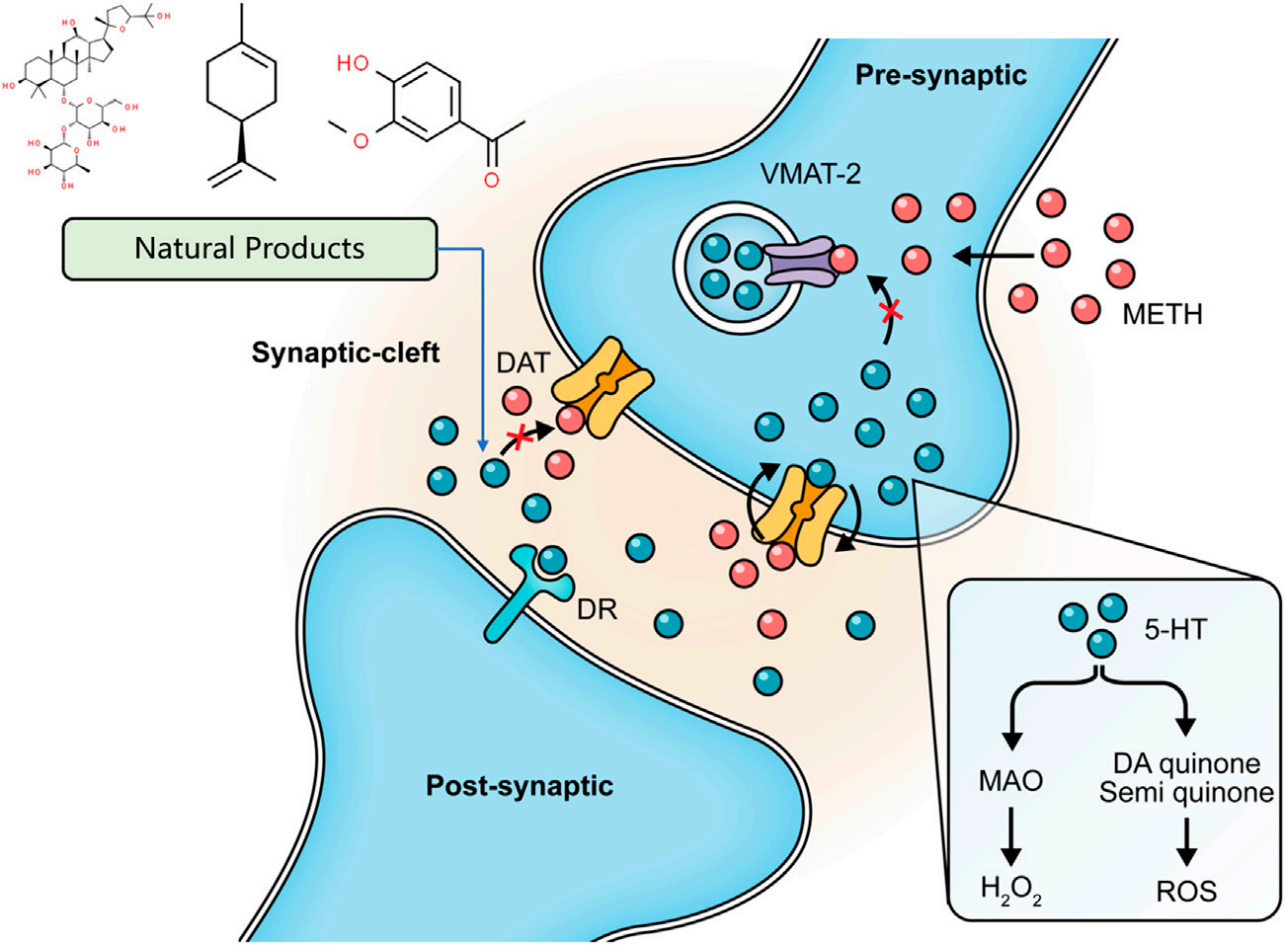
Natural products targeting DA release to attenuate METH-induced apoptosis.

As described above, METH interferes with DA reuptake and causes DA oxidation. Elevated levels of DA readily diffuse in cells and cause oxidative damage, which is closely related to METH-initiated neurotoxic effects. Ginseng total saponin (GTS), an active biological component derived from *Panax ginseng* C. A. Mey., has been reported to be an effective intervention for the central dopaminergic system more than 2 decades ago. Studies conducted in 1994 and 1996 by Kim et al. first identified that GTS could attenuate conditioned place preference in mice, and this effect might be associated with inhibition of METH-induced dopaminergic activation (Kim et al., 1995; Kim et al., 1996). Their follow-up studies demonstrated that GTS pretreatment could partially protect striatal dopaminergic neurons from METH-induced depletions and reduce DA release and its degradation by MAO (Oh et al., 1997a; Oh et al., 1997b). The property of reducing DA release in GTS may alleviate DA autoxidation and ensuing oxidative stress and apoptosis activation. Limonene is a major compound in the essential oils of citrus fruit peels. In 2014, Lin et al. demonstrated that Limonene reduced extracellular DA levels elevated by METH in the nucleus accumbens of rats, which might be resulted from the increase of gamma-aminobutyric acid (GABA) levels (Yun, 2014). In the same year, Miller et al. proposed that subchronic use of Apocynin, a phenolic compound isolated from *Picrorhiza kurroa* Royle ex Benth., could significantly reduce the METH-stimulated DA release in rat striatum (Miller et al., 2014). In 2016, Dang et al. further studied the neuroprotective effects of Apocynin and found that this compound could attenuate mitochondrial dysfunction, microglial activation, and causal apoptotic signaling process through inhibiting the extracellular signal-regulated kinase (ERK)-dependent activation of p47phox (Dang et al., 2016). The major mechanisms of how natural products target DA release to attenuate METH-induced apoptosis are summarized in Figure 1.

### Natural Products Targeting Oxidative Stress and Mitochondria-Dependent Pathway to Modulate METH-Induced Apoptosis

Oxidative stress and mitochondria dysfunction have been considered major mechanisms underlying METH-induced apoptosis. Regulating the generation of ROS and antioxidants may be efficacious strategies to ameliorate mitochondria dysfunction and neural damage. Pseudoginsenoside-F11 (PF11), an ocotillol-type saponin derived from American Ginseng (*Panax quinquefolius L*.) (Li et al., 2000), has been demonstrated to mediate neuroinflammation through decreasing the activation of microglial and the subsequent expression of pro-inflammatory factors and oxidative stress through increasing antioxidant activity (Wang et al., 2014; Zhang et al., 2019). In 2003, a study by Wu et al. showed that PF11 could antagonize the depletion of dopaminergic contents in mice induced by chronic METH administration (Wu et al., 2003). In 2016, Chen et al. further revealed that PF11 could attenuate the METH-evoked oxidative stress in the hippocampus of rats through increasing SOD and GSH-Px activities and decreasing malondialdehyde (MDA) level (Chen. et al., 2016). PF11 also regulated GABAergic neurons and μ-opioid receptors, which are considered to be involved in METH-induced DA release (Fu et al., 2016). *Gastrodia elata* Blume is a well-known herb in traditional Chinese medicine and has often been used to treat various neurological disorders. In early 2011, Shin et al. reported that its methanol extract could significantly attenuate METH-induced ROS in striatal dopaminergic neurons of mice (Shin et al., 2011). Ginsenoside RE, a validated constituent extracted from *Panax ginseng* C. A. Mey. (Liu et al., 2019), showed an antioxidative effect against METH-induced apoptosis. In 2014, Shin et al. proved the mediation effect of Ginsenoside RE for oxidative stress in mitochondria through inhibiting PKCδ (Shin et al., 2014). In 2015, *in vitro* studies by Nam et al. also suggested that Ginsenoside RE could induce the cytosolic and mitochondrial GPx activity and the mitochondrial translocation of cleaved PKCδ (Nam et al., 2015). Resveratrol, a polyphenol found in red wine, has considerable neuroprotective effects, including antioxidation, anti-inflammation, and anti-apoptosis (Howitz et al., 2003). In 2015, Sun et al. reported that it could significantly decrease METH-induced ROS production *in vitro* and the compensatory SOD generation. They also observed that resveratrol might reduce the intracellular Ca^2+^ concentration, suggesting it exerts neuroprotective effects on METH-caused apoptosis through targeting oxidative stress and ER stress (Sun et al., 2015). Another study by Zeng et al. also suggested that the antioxidative effect of resveratrol might attribute to the activation of the keap1-Nrf2 pathway (Zeng et al., 2021). In 2018, Shafahi et al. reported that intervention with crocin, a bioactive constituent of saffron (*Crocus sativus L*.), could regulate the increased SOD and GSH and the ensuing decreased caspase-3 activation in rats stimulated by METH (Shafahi et al., 2018). In 2019, an *in vivo* study by Mozaffari et al. demonstrated that the neuroprotective effects of crocin might be associated with the Cyclic AMP response element-binding protein (CREB) and the Brain-derived neurotrophic factor (BDNF) signaling pathway (Mozaffari et al., 2019). Epigallocatechin gallate (EGCG), a polyphenol compound derived from green tea, is a potent antioxidant. In 2020, Pan et al. observed that EGCG pretreatment could reverse the reduced GPx-4 protein level in the striatum of mice and the overexpression of compensatory SOD-1 protein induced by METH, indicating its effects in preventing METH-caused apoptosis (Pan et al., 2020). Terminalia chebula polyphenol extract (TCPE) is obtained from *Terminalia chebula* Retz. In 2020, Zeng et al. reported that TCPE could protect PC12 cells from METH-elicited DNA damage and apoptosis, which might be related to the inhibition of intracellular ROS and MDA production and the enhancement of SOD and GPx activities (Zeng et al., 2020). Curcumin (CUR) is the main component of turmeric (*Curcuma longa L*.). In 2021, a study by Hadizadeh-Bazaz et al. reported that CUR could increase SOD and GSH and decrease MDA in the hippocampal neurons of mice injected with METH. They also found that CUR could reduce the TNF-α levels to attenuate the neuroinflammation. These effects of CUR contributed to a significant reduction in METH-induced apoptosis (Hadizadeh-Bazaz et al., 2021).

**FIGURE 2:**
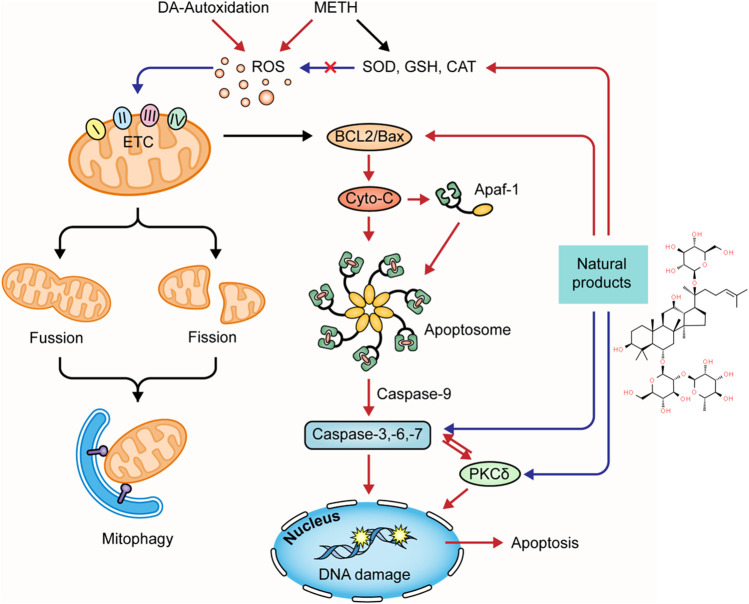
Natural products targeting on oxidative stress and mitochondria-dependent apoptotic pathway to attenuate METH-induced apoptosis.

Several natural products targeting mitochondria-related apoptotic proteins have been explored in recent years. In 2011, Kanthas et al. reported that resveratrol pretreatment could protect dopaminergic cells from MTTH-induced cell death through blocking caspase-3 activation and the subsequent DNA fragmentation (Kanthasamy et al., 2011). Astragaloside IV is the main bioactive component in *Astragalus mongholicus* bunge. In 2015, a study by Sui et al. revealed that the Bcl-2 and caspase-3 were overexpressed in METH-dependent mice, and the former might be a compensatory reaction against apoptosis. Astragaloside IV could enhance the Bcl-2 expression and inhibit the caspase-3 expression and activation (Lu et al., 2015). Asiatic acid is a pentacyclic triterpene extracted from *Centella asiatica* (*L*.) Urb. and possesses multiple bioactivities, including antioxidation, anti-inflammation, and anti-apoptosis. An *in vitro* study conducted in 2017 by Park et al. revealed that Asiatic acid inhibited the cleavage of caspase-3 to reduce the subsequent cleavage of PARP (Park et al., 2017). *Antrodia camphorata* (M. Zang et C. H. Su) Sheng. H. Wu, Ryvarden et T. T. Chang is a medicinal mushroom endemic to Southeast China. In 2017, a study by Chi et al. showed that its enzymes could suppress the METH-evoked mitochondrial apoptosis pathway in PC12 cells by reducing the intracellular ROS levels and inhibiting procaspase-3 cleavage (Chi et al., 2017). In 2020, Yan et al. investigated the anti-apoptotic effect of Gastrodin (GAS), the major bioactive component of *Gastrodia elata* Blume, and reported that GAS intervention could decline the METH-stimulated caspase-3 overexpression in cortical neurons of rats (Yan et al., 2020). An *in vitro* experiment by Ma et al. also observed that GAS could decrease TUNEL-positive cells in the cortical neurons stimulated by METH through modulating CREB/BNDF signaling pathway (Ma et al., 2020). The major mechanisms of how natural products target oxidative stress and mitochondria-dependent apoptotic pathway to attenuate METH-induced apoptosis are summarized in Figure 2.

### Natural Products Targeting Endoplasmic Reticulum Stress-Mediated Pathway to Modulate METH-Induced Apoptosis

**FIGURE 3:**
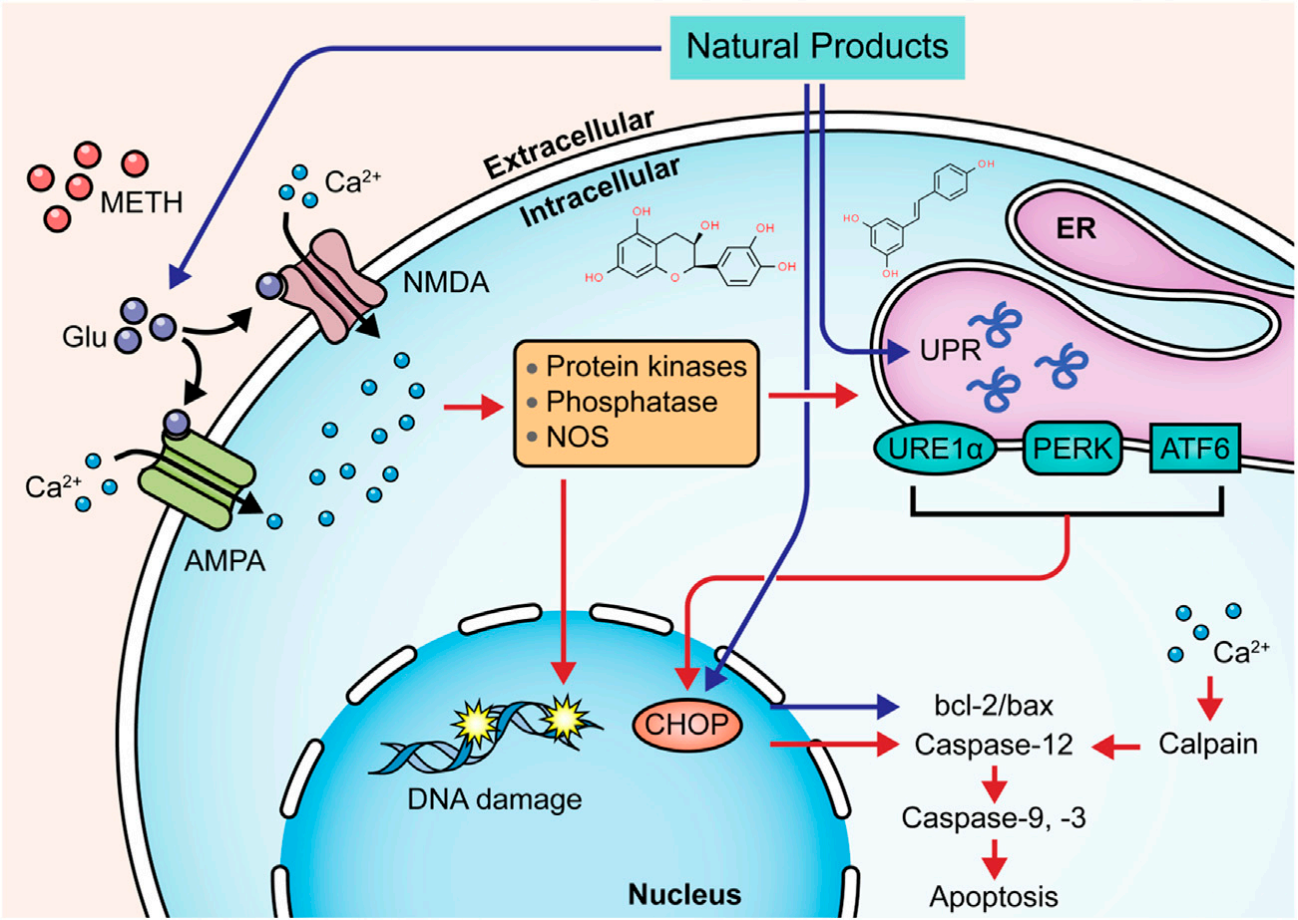
Natural products targeting ERS-mediated pathway to attenuate METH-induced apoptosis.

Targeting the ERS-mediated pathway is also a feasible way for natural products to modulate METH-induced neuronal apoptosis. In 2015, Sun et al. observed that resveratrol might reduce the intracellular Ca^2+^ overload and exert neuroprotective effects on METH-caused apoptosis of dopaminergic neurons through attenuating ERS (Sun et al., 2015). In 2019, Kang et al. reported that Epicatechin, a bioactive flavonoid in green tea, could protect hippocampal neurons from METH-induced cell apoptosis via reducing ERS, and its potential mechanisms might associate with the down-regulation of CHOP, ROS, caspase-3, -8, -9, and PARP (Kang et al., 2019). In 2021, Lee et al. revealed that pretreatment with aromadendrin, a flavonoid obtained from *Abies sibirica* Ledeb. (Syn. *Pinus sibirica* Ledeb), suppressed METH-induced cell death in SH-SY5Y cells by decreasing CHOP and Bax and increasing Bcl-2. It also exhibited a preventative effect on cell apoptosis via restoring the phosphorylation of mTOR to inhibit the PI3K/Akt/mTOR pathway (Lee et al., 2021). The major mechanisms of how natural products target ERS to attenuate METH-induced apoptosis are summarized in Figure 3.

### Natural Products Targeting Neuroinflammation to Modulate METH-Induced Apoptosis

**FIGURE 4:**
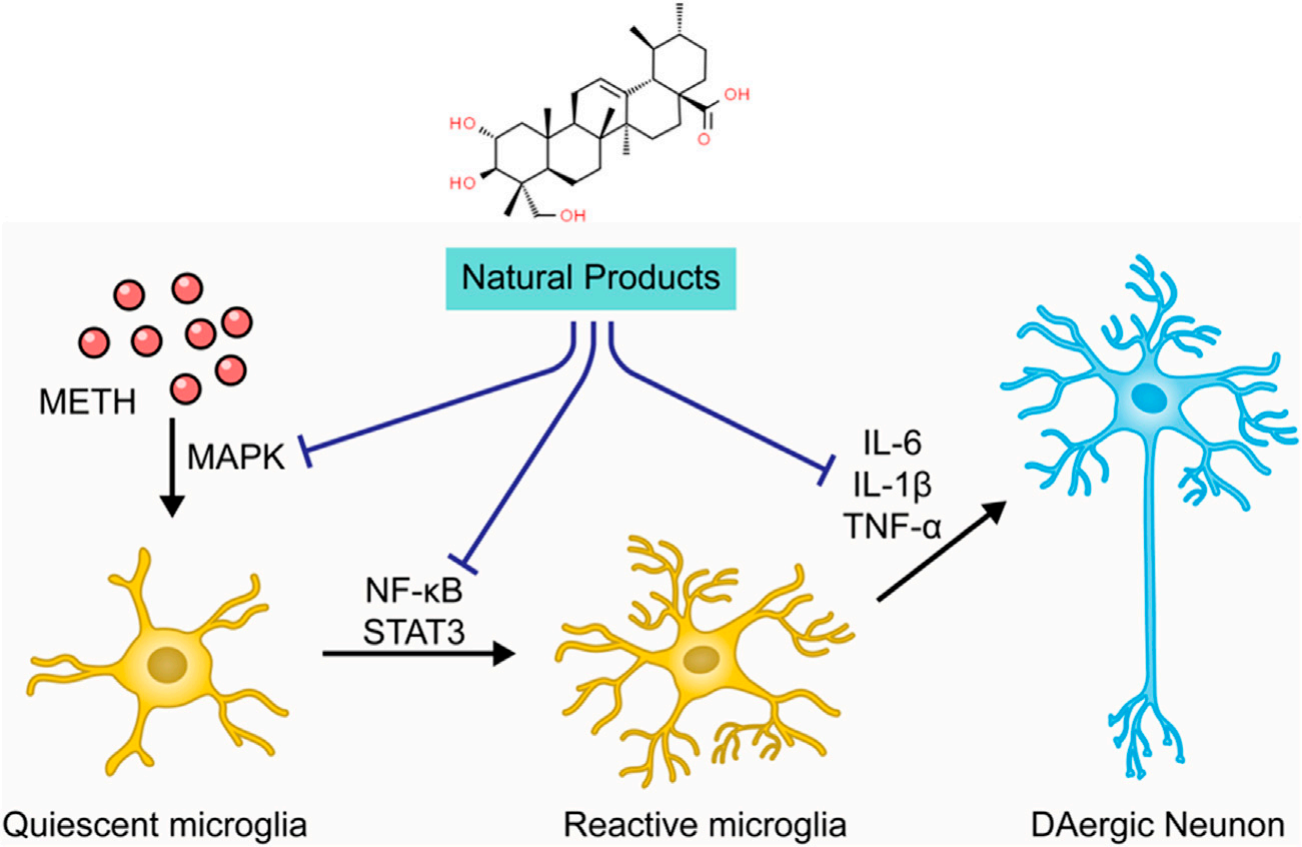
Natural products targeting neuroinflammation to attenuate METH-induced apoptosis.

As stated before, the neuroinflammation induced by activated microglia is crucial in METH-caused neurotoxicity. Accordingly, ameliorating neuroinflammation may be a promising approach to control METH-induced neurotoxicity. In 2014, Shin et al. found that Ginsenoside RE could attenuate the METH-induced activation of microglial in PKCδ (+/+) mice, suggesting that Ginsenoside RE could mediate apoptosis via inactivation of PKCδ (Shin et al., 2014). In 2017, an investigation by Park et al. demonstrated that Asiatic acid could relieve METH-mediated dopaminergic neuroinflammation of SH-SY5Y cells by reducing pro-inflammatory cytokines secretion, such as TNFα and IL-6, and down-regulated the expression of TNF receptor via inhibiting NF-κB, STAT3, and MAPK-ERK signaling pathway. Moreover, Asiatic acid also exerted the inhibitory effects on caspase-3 cleavage and the subsequent PARP cleavage and the mediative effects on Bcl-2 and Bax (Park et al., 2017). In addition, in 2018, Shafahi et al. reported that crocin treatment could reduce the level of TNF-α in the hippocampus of rats stimulated by METH, suggesting that crocin could also exert an antiapoptotic effect by mediating METH-induced neuroinflammation (Shafahi et al., 2018). The major mechanisms of how natural products target neuroinflammation to attenuate METH-induced apoptosis are summarized in Figure 4.

## Conclusion and Perspectives

Most of the METH-elicited neuropsychiatric consequences are attributable to its neurotoxicity, including direct damage to central dopaminergic and serotonergic terminals and the subsequent neurodegenerative changes upon the activation of neuronal autophagy, apoptosis, and necrosis (Davidson et al., 2001; Yu et al., 2015; Sanchez et al., 2018). In recent years, important progress has been achieved in interpreting the complex mechanism underlying METH-produced neurotoxicity. Neuronal apoptosis has been considered the main pathogenic factor in METH-induced neurodegeneration. Animal studies have confirmed that pro-apoptotic changes in the brain are highly associated with METH-induced neuropsychiatric symptoms, such as locomotor sensitization, psychosis, memory impairments, and cognitive impairments (Shin et al., 2017). With the decrease of central monoaminergic terminals and the depletion of dopamine and serotonin, these psychiatric complications are sustained (Rusyniak, 2013), leading to failed anti-addiction treatments, relapse to drug use, and increased risk of various neurodegenerative diseases (Shukla and Vincent, 2020). In view of the devastating societal and public health burdens caused by METH abuse, further in-depth explorations in pharmacotherapies are highly anticipated. In this review, we summarized and analyzed these natural products that have been proven to possess significantly *in vitro* and *in vivo* protective effects against METH-induced neuronal apoptosis through mediating multiple pro-apoptotic factors, such as DA release, oxidative stress, mitochondria-dependent apoptotic pathway, ERS-mediated apoptotic pathway, and neuroinflammation.

Notwithstanding the numerous studies conducted to excavate the effects of natural agents in the prevention and treatment of METH-caused neuronal apoptosis, the discovery and development of novel and effective novel pharmacotherapies still require more sophisticated works in both preclinical and clinical investigations. First, METH abuse involves the activation of multiple apoptotic pathways. However, current research has primarily focused on using a single natural agent to block a single pathway. Given the complex nature of METH-initiated neurotoxicity, strategies that combine multiple natural compounds and target multiple pathways may yield more encouraging medications. Interestingly, the protective effect of certain natural products, such as Crocin, Resveratrol, Curcumin, and Apocynin, on METH-evoked apoptosis have been attributed to the regulation of multiple targets simultaneously. From the perspective of optimal “cocktail” drug design, they may yield a promising future in novel anti-METH strategies (Radhakrishnan and Tidor, 2008). Approaches combining systems biology with traditional computational-aided drug design are applicable for developing drug “cocktail” regimens to modulate the core target network of METH. Second, the available studies have focused on the effects of natural products on oxidative stress, and the interpretation of their actions on other pathogenic factors is still insufficient. Several natural agents have demonstrated promising potential to regulate neuronal apoptosis in other fields, including neurodegenerative diseases (Kim and Kim, 2018), which indicate that discovery and exploration of more potential natural candidates can be anticipated.

Third, the specific molecular and genetic mechanisms underlying the anti-apoptotic effects of natural products are still elusive. The safety of natural products and their interaction with conventional drugs have become major concerns. Therefore, further studies on their pharmacokinetics, pharmacodynamics, and toxicological profiles are of great necessity (Jha and Rathi, 2008; Nauffal and Gabardi, 2016; Gaston et al., 2020). Follow-up studies need to be performed to evaluate the potential toxicity of the natural agents. In addition, no clinical trial regarding the anti-apoptotic effects of natural products for METH-induced neuronal apoptosis has been documented, and future studies may devote more efforts to designing high-quality and well-designed clinical trials to verify their therapeutic effects and explore the relative mechanism. Lastly, new and emerging insightful scientific findings on METH-induced neuronal apoptosis have continually updated our understanding of its biomechanisms. For example, recent studies confirmed that CCAAT/enhancer-binding protein (C/EBP-β), Lipocalin2 (lcn2), and Sigma-1 receptor (σ-1R) might play an important role in METH-induced microglial apoptosis (Shen et al., 2016; Chen et al., 2019). These new findings may provide new directions for future studies of natural agents and yield more refined and robust evidence. In addition, it is also worth noting that except for the natural products stated above, other agents, such as Vitamin C (L-ascorbate) and Melatonin, also have the potentials to be developed as candidate anti-apoptotic drugs in clinical management (Huang et al., 2012; Nguyen et al., 2015).

In conclusion, this review summarized and analyzed these natural products with anti-apoptotic properties to prevent and treat METH-induced neurotoxicity by modulating DA release, oxidative stress, mitochondria dysfunction, caspase cascades, and neuroinflammation. Hopefully, this review may provide helpful information for their further exploration preclinically and clinically.
